# Bibliography

**Published:** 1897-03

**Authors:** 


### Bibliography.

The Items of Interest for January, 1897, is certainly a new departure in dental journalism, and the editor, Dr. R. Ottolengui, deserves to receive congratulations not only for the life he has succeeded in infusing into this trade journal, but for the somewhat original conceptions connected with it. If success comes in the truest and best sense it will not be through the influence of flaring heads and subheads, but by a higher and broader conception of what is truly needed in dentistry.

The "Items of Interest" has heretofore catered to an element in dentistry that regarded condensed statements as all sufficient. If the present editor can induce an enlarged view of scientific study he will have accomplished a great work. The Items in its improved form cannot yet be regarded as the "best dental journal" nor even the best trade journal, but it comes nearer that than the former.

The dental public is not particularly interested to know whether these improvements in the "Items of Interest" cost five thousand dollars per year more than heretofore, but it should be interested to have an addition to dignified journalism, but whether this will meet that want remains for future numbers to demonstrate.

Dental Materia Medica, Pharmacology, and Therapeutics. By Charles W. Glassington, M.R.C.S., L.D.S., Edinburgh, Senior Dental Surgeon, Westminster Hospital, etc. London: J. & A. Churchill, 1896.

This book, while in one sense a compend, has the merit not possessed by all such works of being concise, and at the same time supplying, if not all the information required, sufficient for the average student to memorize.

For a small book it is admirably arranged, omitting much that is superfluous in the study of Materia Medica, while at the same time it gives ample scope to matters that require special attention in dentistry. The therapeutic division is generally quite full, and there is much throughout the book that deserves special praise, and but little to condemn.

The following advice in the writing of prescriptions is worthy of being followed by those who delight in complicated formulæ: "Some drugs, when properly combined, materially assist the action of others; but if one is *certain* of the action of a single drug, by all means use it without combination."

It is not to be expected that a book of this size would or could give the authority for each statement, but omission in this respect leads to error. In the use of chlorinated lime for bleaching teeth the entire process is given without credit to the one originating it.

A very excellent chapter on "General Anæsthesia for Dental Operations," by James Maughan, M.B., London, anæsthetist, National Dental Hospital, closes this volume, which can be recommended as superior to most of its kind and valuable to the student as a book of reference.

